# Stereotactic radiotherapy of nodal oligometastases from prostate cancer: a prisma-compliant systematic review

**DOI:** 10.1007/s10585-022-10183-6

**Published:** 2022-08-18

**Authors:** Alice Zamagni, Mattia Bonetti, Milly Buwenge, Gabriella Macchia, Francesco Deodato, Savino Cilla, Erika Galietta, Lidia Strigari, Francesco Cellini, Luca Tagliaferri, Silvia Cammelli, Alessio Giuseppe Morganti

**Affiliations:** 1 Department of Experimental Diagnostic and Specialty Medicine (DIMES), Alma Mater Studiorum - Bologna University, Bologna, Italy; 2 Radiation Oncology Unit, Gemelli Molise Hospital - Università Cattolica del Sacro Cuore, Campobasso, Italy; 3 Istituto di Radiologia, Università Cattolica del Sacro Cuore, Rome, Italy; 4 Medical Physics Unit, Gemelli Molise Hospital - Università Cattolica del Sacro Cuore, Campobasso, Italy; 5 Medical Physics Unit, IRCCS Azienda Ospedaliero-Universitaria di Bologna, Bologna, Italy; 6 Dipartimento di Diagnostica per Immagini, Radioterapia Oncologica ed Ematologia, Fondazione Policlinico Universitario “A. Gemelli” IRCCS, Radioterapia Oncologica ed Ematologia, Rome, Italy; 7 Dipartimento Universitario Diagnostica per Immagini, Radioterapia Oncologica ed Ematologia, Università Cattolica del Sacro Cuore, Rome, Italy; 8 Radiation Oncology, IRCCS Azienda Ospedaliero-Universitaria di Bologna, Bologna, Italy

**Keywords:** Prostate cancer, Lymph node metastasis, Stereotactic radiotherapy, Systematic review

## Abstract

Androgen deprivation therapy (ADT) is the standard treatment of metastatic prostate cancer (PCa). However, metastases-directed therapies can delay the initiation or switch of systemic treatments and allow local control (LC) and prolonged progression-free survival (PFS), particularly in patients with lymph nodes (LN) oligometastases. We performed a systematic review on stereotactic body radiotherapy (SBRT) in this setting. Papers reporting LC and/or PFS were selected. Data on ADT-free survival, overall survival, and toxicity were also collected from the selected studies. Fifteen studies were eligible (414 patients), 14 of them were retrospective analyses. A high heterogeneity was observed in terms of patient selection and treatment. In one study SBRT was delivered as a single 20 Gy fraction, while in the others the median total dose ranged between 24 and 40 Gy delivered in 3–6 fractions. LC and PFS were reported in 15 and 12 papers, respectively. LC was reported as a crude percentage in 13 studies, with 100% rate in seven and 63.2–98.0% in six reports. Five studies reported actuarial LC (2-year LC: 70.0–100%). PFS was reported as a crude rate in 11 studies (range 27.3–68.8%). Actuarial 2-year PFS was reported in four studies (range 30.0–50.0%). SBRT tolerability was excellent, with only two patients with grade 3 acute toxicity and two patients with grade 3 late toxicity. SBRT for LN oligorecurrences from PCa in safe and provides optimal LC. However, the long-term effect on PFS and OS is still unclear as well as which patients are the best candidate for this approach.

**Supplementary Information:**

The online version contains supplementary material available at 10.1007/s10585-022-10183-6.

## Introduction

Prostate cancer (PCa) is the second most frequent cancer and the fifth leading cause of cancer death in men worldwide [1]. In Developed Countries, one out of eight men will be diagnosed with PCa during their lifetime [2]. PCa incidence and death rates are strictly related to the widespread use of PSA screening since it allows early tumor detection but also increases the identification of latent PCa [1]. Moreover, advances in imaging techniques in recent years led to increased detection of oligometastatic PCa and thus to a growing interest in metastases-directed therapies (MDT) [3].

The optimal treatment in this setting is still under debate due to a lack of strong evidence. Moreover, based on international guidelines [4, 5], the current treatment standard for metastatic PCa is still androgen deprivation therapy (ADT) (± other systemic therapies), without specific indications for the subset of oligometastatic patients. However, increasing evidence suggests that a more targeted management of oligometastatic PCa could play a role as a “curative” option in the multimodal treatment approach [6] with high local control (LC) rate and delay of systemic treatments. As a result, 75% of the Advanced Prostate Cancer Consensus Conference (APCCC, 2019) panelists recommended systemic therapy *plus* local treatment of all lesions for most patients with oligorecurrent PCa [7] due to better tolerability of MDT [7–11] compared to chemotherapy or ADT [12, 13].

Even though publications in this setting have increased in the last years, at least two questions are still open, namely, what is the impact of MDT on overall survival (OS) and cancer-specific survival and how to select patients suitable for this approach. For patient stratification, following the recent classification proposed by the European Society for Radiotherapy and Oncology (ESTRO) and Radiation Therapy Oncology Group (RTOG) consensus [14], a first distinction should be made between synchronous and metachronous oligometastatic PCa [15, 16]. In fact, metachronous nodal oligometastases should be considered as a potentially different entity compared to bones or visceral oligometastases (or at least as a different step of disease progression) [17, 18] being lymph nodes (LN) oligometastases a favorable subset in terms of disease progression [17, 19, 20].

However, clear evidence (especially from randomized phase III trials) in this setting is lacking. Therefore, we performed a systematic review to summarize the available results on stereotactic body radiotherapy (SBRT) as MDT in nodal oligometastases from PCa.

## Materials and methods

The protocol of this systematic review was submitted to the PROSPERO international prospective register of systematic review on August 25^th, 2020^ [21]. The Preferred Reporting Items for Systematic Reviews and Meta-Analysis (PRISMA) guidelines were followed to perform the analysis [22]. We searched for articles reporting on the outcome of metachronous oligometastatic PCa patients treated with SBRT for LN metastases. The primary objectives of the review were LC and progression-free survival (PFS). We also collected data on the biochemical response (BRes), biochemical relapse, clinical response (CRes), androgen deprivation therapy-free survival (ADT-FS), OS, and toxicity when reported with at least one of the primary endpoints.

### Bibliographic search

A literature search for relevant studies was conducted in PubMed, Scopus, and Cochrane library up to July 1st, 2021, using the combination of several terms like: “lymph node”, “metastases”, “stereotactic body radiotherapy”. The term “prostat*” was not included in the search criteria to allow the identification of papers reporting data on mixed primary tumors. The complete search strategy is reported in Appendix 1. The reference list of the selected papers was checked to eventually identify additional manuscripts. Only studies published in English were included.

### Inclusion criteria

We used the Population, Intervention, Comparator, Outcome and Study design (PICOS) approach to assess study eligibility. We included studies on PCa patients with metachronous oligometastatic disease (synchronous oligometastatic disease diagnoses were not allowed) limited to the LN and treated with SBRT (max 10 fractions). Papers were excluded if patients were treated with SBRT as concomitant or sequential boost combined with elective nodal irradiation (ENI) or in the primary treatment setting (unless the latter case involved a small minority of the patients' cohort). Papers should report at least one of the two selected primary endpoints: LC or PFS (both actuarial and crude rates allowed). If available, other selected outcomes were collected. Studies involving also patients treated with therapies other than SBRT were included, but only if the primary endpoints of patients treated with SBRT on LN oligometastases from PCa were separately reported. Moreover, studies reporting duplicated data were excluded and studies reporting partially duplicated data were excluded if the outcome was not reported separately for duplicated and non-duplicated data. We also excluded systematic or narrative reviews, meta-analysis, guidelines, studies on animal models, preclinical studies, study protocols, case reports, surveys, and planning and imaging studies.

### Study selection

Studies were independently screened by AZ and MBo at the title and abstract level, and duplicate publications were removed. After this screening, papers considered suitable for our analysis were examined at full-text level to select articles eligible for the systematic review (**Appendix 2**). Any discrepancies during the selection process were discussed and eventually resolved by a third author (AGM).

### Data extraction

Data from the selected papers were independently extracted by AZ and MBo and collected in a predefined form. In the event of conflicting data, the final decision was discussed with the participation of AGM. The following information was abstracted from the selected papers: authors, year of publication, reference, study design, enrollment period, number of patients, number of treated LN, patients age, imaging modality, hormonal status, PSA at recurrence, selection criteria for patients inclusion, follow-up (FU) duration, the time between primary treatment and SBRT, SBRT details (total dose, number of fraction, SBRT delivery technique), use of concomitant and/or adjuvant ADT, outcomes in term of LC, PFS, BRes, biochemical relapse, CRes, ADT-FS (both as crude and actuarial rate), and acute and late toxicity.

## Results

### Search results

**Fig. 1:**
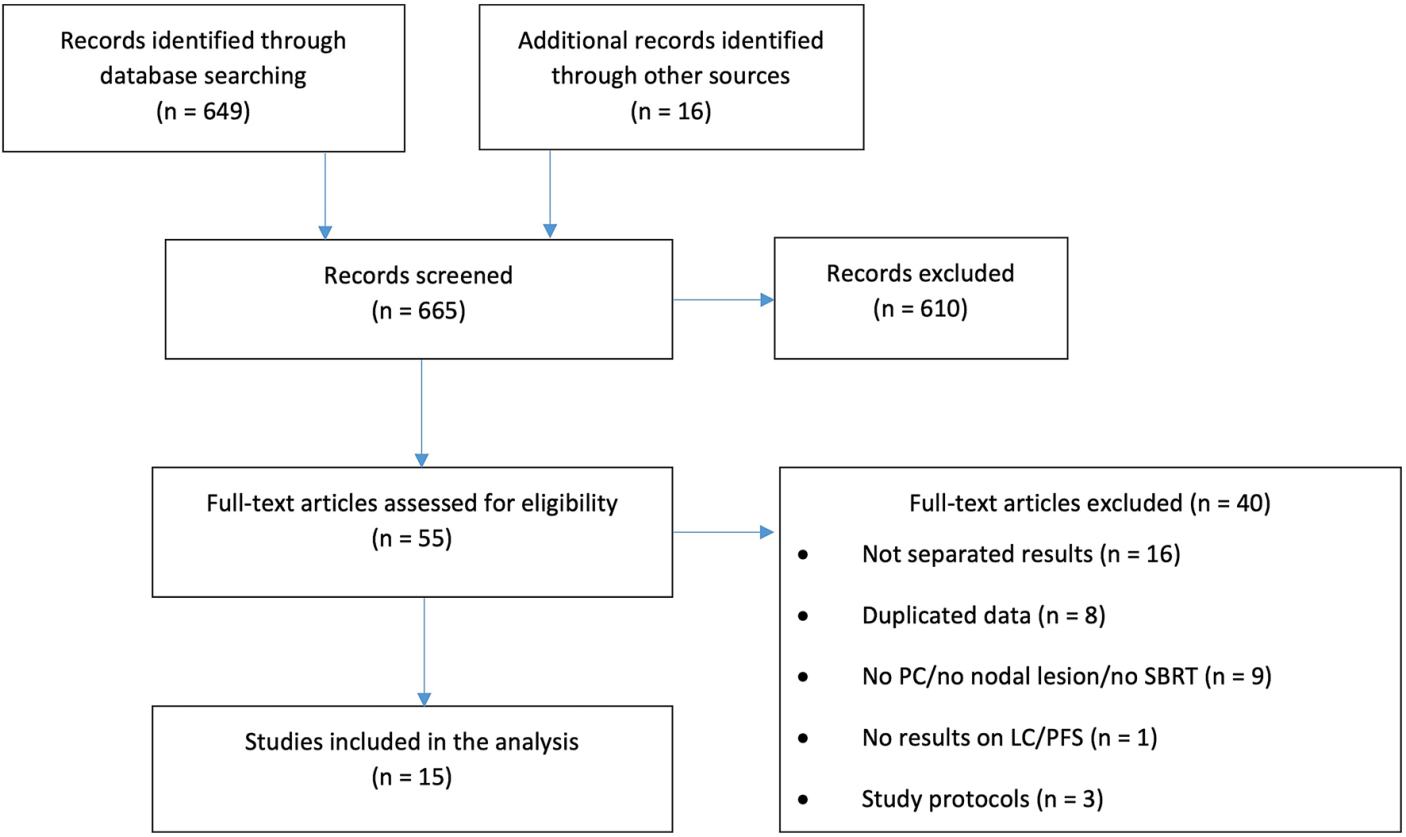
Prisma flowchart describing the selction of studies

Figure 1 shows the flowchart of study selection. A total of 665 studies were initially identified. After the title-abstract screening, 55 full-text articles were examined (Appendix 2), and 15 papers were included in the final analysis. All but one were observational case series: three were prospective [23–25] and 11 were retrospective case series [26–36]. The only interventional trial was a phase II study [37]. All selected studies included only patients with metastatic PCa. Three studies reported only LC [24, 34, 35], while 12 studies reported both LC and PFS [23, 25–33, 36, 37]. Other included outcomes were BRes, biochemical relapse and CRes, reported in four [27, 29, 30, 35], four [30, 32, 33, 35] and two papers [26, 32], respectively. Toxicity was reported in nine papers [24, 25, 27–33], ADT-FS was reported in three studies [29, 33, 35] and OS was reported in three studies [26, 31, 33]. Both toxicity and ADT-FS were reported for the entire cohort, including metastases other than nodal, in four studies [23–25, 34, 37].

### Patients and tumor characteristics

Overall, the analyzed studies included 414 patients with LN metastases *plus* 10 patients with both LN and bone metastases from PCa (Table 1). Particularly, in seven studies [23–25, 31, 34, 36, 37] the patients population was heterogeneous due to the inclusion of patients with LN and/or bone metastases. In these studies, the percentage of patients with LN metastases ranged from 39.4% to 85.0% (median: 63.5%). The median number of patients per study, considering only patients with LN metastases, was 25 (range 7–94) while the median number of treated LN per study was 34 (range 8–124). In three studies [23, 25, 26] the total number of treated LN was not specified. Notably, only one study [29] reported results on more than 50 patients and more than 50 lesions, while six studies reported results on less than 20 patients [24, 27, 28, 31, 36, 37].

**Table 1:** Studies characteristics

Author, year	Study design	Enrollment period	Total patients number§	Treated patients/lesions	Pts with LN and extra-nodal disease	LN treated per patient	Diagnostic imaging	Hormonal status	PSA at recurrence, median (range) [ng/ml]	Selection criteria	FU median/mean (range) [months]	pts age, median (range) [years]	Time between primary treatment and SBRT, median (range) [months]
Jereczek-Fossa et al., 2009 [28]	RC	12/2003–02/2008	14	7/8#	0	1 LN: 85.7% 2 LN: 14.3%	choline PET	NR	3.5 (0.8–11.6)	NR	18.9 (9.2–42.4)	mean 71.7	58.6 (25.5–149)
Casamassima et al., 2011 [26]	RC	NR	25	25/NR	0	NR	choline PET	NR	5.65 (0.37–181.6)	isolated LN recurrence	29 (14.4–48)	NR	NR
Jereczek-Fossa et al., 2012 [27]	RC	05/2007–12/2009	34	12/16	0	1 LN: 66.7% 2 synchronous LN: 8.3% 2 LN metachronous: 25.0%	choline PET	NR	1.77 (0.22–15.5)	at least 24 mo between primary treatment and recurrence	(4.3–35.4)	NR	> 24 mo
Decaestecker et al., 2014 [25]	PC	05/2005–10/2013	50	27/NR	0	NR	choline PET, FDG PET	hormone naive/ sensitive	NR	no ADT at recurrence	NR	NR	NR
Detti et al., 2015 [30]	RC	11/2011–12/2013	30	30/39	0	1 LN: 70.0% 2 synchronous LN: 16.7% 2 metachronous LN: 13.3%	choline PET	NR	NR	NR	12 (2–24.9)	NR	75.6 (4.8–222)
Napieralska et al., 2016 [31]	RC	12/2011–03/2014	18	18*/31	2 (11.1%)°	1 LN: 50.0% 2–4 LN: 50.0%	66.6% choline PET, 16.7% CT, 16.7% MRI	NR	4.66 (0.01–15.58)	≤ 5 lesions, LN and extranodal admitted	15.6 (0–33)	median 69 (58–80)	46 (0–149)
Pasqualetti et al., 2016 [24]	PC	NR	29	17*/25	2 (11.7%)	NR	NR	NR	NR	≤ 3 lesions	NR	NR	NR
Bouman-Wammes et al., 2017 [34]	RC	01/2009–12/2015	43	34*/41	1 (2.9%)	NR	choline PET	hormone sensitive	NR	≤ 4 lesions, no ADT at recurrence	NR	NR	NR
Franzese et al., 2017 [32]	RC	2007–2015	26	26/38	0	1 LN: 61.5% 2–4 LN: 38.5%	choline PET	NR	3.3	≤ 4 lesions	29.4 (2.9–79.5)	70 (56–90)	NR
Ingrosso et al., 2017 [33]	RC	09/2008–12/2014	40	40/47	0	1 LN: 82.5% 2 synchronous LN: 2.5% 2 metachronous LN: 15.0%	choline PET	NR	4.2 (0.44–17.9)	isolated LN recurrence	23.8 (3.73–79.8)	74 (58–83)	37.45 (11.16–216.03)
Jereczek-Fossa et al., 2017 [29]	RC	05/2012–10/2015	94	94/124	0	1 LN: 74.5% 2 LN: 20.2% 3 LN: 4.3% 4 LN: 1.0%	95.8% choline PET, 3.1% MRI 1.1% CT	NR	3.5	≤ 5 lesions	18.5 (3–42)	70.7 (IQR 65–76)	49.6 (27.2–122.4)
Kneebone et al., 2018 [23]	PC	11/2014–07/2016	57	39*/NR	2 (5.13%)	1 LN: 69.2% 2–3 LN: 25.6%	PSMA PET	hormone naive/ sensitive	NR	≤ 3 lesions (LN or bone), no ADT at recurrence	NR	NR	NR
Siva et al., 2018 [37]	phase II study	04/2013–11/2014	33	13/15	1 (7.7%)	1 LN: 92.3% 3 LN: 7.7%	NaF PET	hormone sensitive/ castration resistant	6.4	≤ 3 lesions	24	NR	NR
Oehler et al., 2019 [35]	RC	01/2010–01/2015	25	25/36	0	1 LN: 64.0% 2 LN: 28.0% 3 LN: 8.0%	choline PET	hormone naive	mean 3.49 (1.02–14.54)	≤ 3 lesions, hormone-naive	18 (IQR: 14–22)	mean 68 (52–81)	NR
Ong et al., 2019 [36]	RC	01/2016-NR	20	17/21	2 (11.7%)	NR	PSMA PET	NR	NR	≤ 3 lesions, no ADT at recurrence	15.9 (6.7–35.5)^	67^	34 (5–127)^

*ADT* androgen deprivation therapy, *FU* follow-up, *IQR* interquartile range, *LN* lymph node(s), *NR* not reported, *PC* prospective cohort study, *PET* positron emission tomography, *RC* retrospective cohort study

^§^The number includes pts with extranodal disease (entire cohort)

*The number includes pts with both LN and bone metastases

°2 pts had extranodal metastases associated with LN metastases and were treated with chemotherapy

^#^7 pts treated with cyberknife are also included in Jereckzek-Fossa 2012 (25) and therefore are not evaluated between the results of this paper

^Data are reported even if referred to the whole cohort since 85% of pts where treated for LN

In 10 studies [24, 26–33, 36] the patients hormonal status (hormone-naïve, hormone-sensitive, castration-resistant) was not specified, while one study focused on hormone-naïve patients [35], two on hormone-naïve and hormone-sensitive patients [23, 25], one on hormone-sensitive patients [34], and one on hormone-sensitive and castration-resistant patients [37]. The enrollment period was reported in all but three studies [24, 26, 36] and ranged from 2003 and 2016 with a median duration of 3.8 years (range 1.6–8.9 years) [23, 25, 27–35, 37]. In only two studies the enrollment period was shorter than two years [23, 37]. The site of treated LNs was specified in 10 papers [25–29, 32, 33, 35–37], and not reported in five studies [23, 24, 30, 31, 34]. Most papers reported data on the International Society of Urological Pathologists (ISUP) risk group and/or on the Gleason Score (GS) of the primary tumor. Particularly, two studies reported the ISUP risk group [27, 30], three studies reported the GS [26, 29, 31] and two studies reported both [33, 35]. In six studies [23, 25, 27, 34, 36, 37] this information was reported for the whole cohort but not specified for patients with LN metastases. The primary treatment of PCa was reported in seven studies [26, 28–31, 33, 35] and not reported in two studies [24, 32] while in six studies it was reported only for the entire patients cohort [23, 25, 27, 34, 36, 37]. Only three studies reported data on any primary treatment of regional LN [30, 31, 35].

Follow up duration was reported in all studies; in four of them it was reported for the entire cohort of patients only [23–25, 34], while in 11 it was specifically reported for LN metastases [26–33, 35–37]. In the latter group, the median follow up time ranged between 12.0 and 29.4 months (median: 18.9 months). Only 3 studies had a median follow up of at least 2 years [26, 32, 37]. Median time between primary treatment and SBRT for metachronous LN metastases was reported in seven studies [27–31, 33, 36], ranging between 34.0 and 75.6 months (median: 46.0 months). Three studies enrolled only patients with a time interval between primary treatment and LN recurrence of at least 24 months [27–29].

The oligometastatic status was confirmed in most studies using [18F] Choline-PET/CT [26–35], in one using [18F] Choline or [18F] FDG-PET/CT [25], in one using [18F]NaF-PET/CT [37] and in two using PSMA-PET/CT [23, 36]. One study [24] did not report the imaging technique used for staging confirmation.

### Stereotactic body radiation therapy

Data on dose and fractionation were reported in all studies and are summarized in Table 2. SBRT was delivered in a single fraction by Siva et al. [37] while in three studies [24, 30, 33] only a small percentage of patients were treated with this schedule. When reported [26–29, 31–33, 35], median dose ranged between 24 and 40 Gy (median: 30 Gy) in 3–6 fractions (median: 3 fractions). SBRT was delivered with Cyberknife (CK), Volumetric Modulated Arc Therapy (VMAT) technique or both in one [29], four [27, 30, 31, 35], and eight studies [23–26, 28, 32, 34, 36], respectively. In two studies the SBRT technique was not specified [33, 37].

**Table 2:** Radiation treatment characteristics

Author, year	Dose (Gy)/Fractions	GTV to PTV expansion	RT delivery technique	SBRT combined to ENI	ADT combined to SBRT, median duration
Jereczek-Fossa et al., 2009 [28]	20–45/2–3 fr	5–9 mm (anisotropic)	VMAT	NO	57.1%, mean 15 mo
Casamassima et al., 2011 [26]	30/3	5 mm	VMAT	28.0%*	NR
Jereczek-Fossa et al., 2012 [27]	33/3	1–2 mm	CK	NO	75%, 17.5 mo
Decaestecker et al., 2014 [25]	30–50/3–10	3 mm	IMRT/VMAT	NO	NO§
Detti et al., 2015 [30]	24–36/1–5	2 mm	CK	NO	33.3%
Napieralska et al., 2016 [31]	24–45/1–3	4–5 mm	CK	11.1%	100%
Pasqualetti et al., 2016 [24]	24–27/1–3	3 mm	VMAT	NO	NR
Bouman-Wammes et al., 2017 [34]	30–45/3–5	3–5 mm	VMAT	NO	NO
Franzese et al., 2017 [32]	25–45/4–6	5 mm	VMAT	NO	57.7%
Ingrosso et al., 2017 [33]	12–50/1–5	5–8 mm	NR	NO	47.5%
Jereczek-Fossa et al., 2017 [27]	15–36/3–6	2–3 mm	10.6% CK, 89.4%VMAT	NO	36.2%, 14.5 mo°
Kneebone et al., 2018 [23]	PTV HD: 30–50/3–5 PTV LD: 24–30/3–5	PTV HD: GTV + 5 mm PTV LD: CTV LD + 10 mm	VMAT	NO	NO
Siva et al., 2018 [37]	20/1	5 mm	NR	NO	NR
Oehler et al., 2019 [35]	30–45/3	2–4 mm	CK	NO	NO
Ong et al., 2019 [36]	35–40/5	5 mm	VMAT	NO	NO

*50 Gy in 25 fr ENI + boost on positive LN 24 Gy in 3 fr

^§^Until 05/2012 pts were treated with single injection of short acting LHRH analogue concomitant with SBRT as radiosensitizer

°1.1% (1 patient) treated with taxane-based chemotherapy

Dose specification was not clearly reported in seven papers [23, 24, 29, 30, 32, 34, 35], while dose was prescribed to a defined isodose line [25–27, 31, 33, 36, 37] or to the isocenter [28] in seven studies and in one report, respectively. Only one study reported the Gross Tumor Volume (GTV) and the Planning Tumor Volume (PTV) size (mean: 6.6 cc and 25.0 cc, respectively) (31). Treatment margins were reported in all studies: the Clinical Target Volume (CTV) or GTV to PTV margin ranged between 1 and 8 mm [23–37]. In all but one study [28] the applied margin was isotropic. Notably, Kneebone et al. [23] used a Simultaneous Integrated Boost (SIB) technique with two volumes treated at different dose levels: GTV + 5 mm was defined as the high dose PTV, while CTV low risk (nodal chain of involved LN) *plus* 1 cm was defined as the low dose PTV. Finally, Casamassima et al. [26]and Napieralska et al. treated 28.0% and 11.1% of patients with ENI *plus* SIB on PET-positive LN, respectively.

### Androgen deprivation therapy

Most papers did not report detailed data on ADT prescription at the time of primary diagnosis [23–30, 32, 33], while Napieralska et al. [31] reported adjuvant ADT in the majority of patients (88.9%) and Bouman-Wammes et al. and Ong et al. in a small percentage of subjects (14.7% and 15.0%, respectively) [34, 36]. Finally, Oehler et al. treated a cohort of hormone-naive patients [35]. More data were available on ADT prescription after oligorecurrence diagnosis. Information on the percentage of patients in whom ADT was prescribed before SBRT was available in two studies [30, 33], while in one it was reported for the whole cohort [37]. In seven studies [27–33] ADT was prescribed concurrently with SBRT to 33.3–100% of patients. When specified, the median duration of ADT ranged between 14.5 and 17.5 months. Decaestecker et al. [25] used a single injection of short-acting LH-RH analog concurrent to SBRT until May 2012. In four series [23, 34–36], concomitant ADT was not prescribed to any patient since it was an exclusion criterion of the study. Finally, in three papers [24, 26, 37] data on ADT prescription was not available.

### Evaluation modalities

#### Local control

LC was generally defined as “freedom from in-field progression” in most studies [23, 24, 26–30, 32, 33], while six studies provided a more specific definition of the “in-field area” (i.e. progression in the “PTV area” [25, 35]or in the “area within the 20% isodose line” [36]or in the “high dose radiation volume” [34]). Only three studies reported the specific definition of LC evaluation criteria (i.e., RECIST criteria [31, 37] or local PSMA-avid disease progression [36]). Three studies [29, 31, 32] reported both crude actuarial LC rates. LC was reported only as crude rate in 10 studies [23–25, 27, 28, 30, 33–36] and only as actuarial result in two studies [26–37]. Actuarial LC was reported at 1-, 2-, and 3-year in three [26, 31, 32], five [26, 29, 31, 32, 37], and two [26, 32] studies, respectively (Table 3).

**Table 3:** Primary outcomes

Author, year	Criteria and imaging used during the follow-up	Local Control			Progressive Disease	Progression-free Survival	
		Definition	Crude rates	Actuarial rates	Definition	Crude rates	**actuarial** **rates**
Jereczek-Fossa et al., 2009 [28]	BF: any increase over PSA pre-SBRT or PSA > PSA nadir + 0.1	freedom from in-field progression	100%	NR	Biochemical and/or clinical failure	42.9% (only clinical failures registered)	NR
Casamassima et al., 2011 [26]	choline PET 60 days after SBRT and then periodically	freedom from in-field progression	NR	1y: 95% 2y: 90% 3y: 90%	NR	56.5%	1y: 80% 2y: 50% 3y: 17%
Jereczek-Fossa et al., 2012 [27]	BF: PSA increase > 10% from pre-SBRT value- > choline PET	freedom from in-field progression	100%	NR	Biochemical and/or clinical failure	68.8% (only clinical failures registered)	2.5y: 63.5%
Decaestecker et al., 2014 [25]	BF: 3 rising PSA after initial response or PSA rise above pre-SBRT PSA- > FDG/choline PET	freedom from progression within irradiated PTV	100%	NR	Absence of new mets and/or progression of untreated mets	37.5% (24 evaluable pts with pelvic LN mets), (62.5% PD: 29.2% pelvic LN, 12.5% extra pelvic LN, 20.8% bones)	NR
Detti et al., 2015 [30]	CT scan/MR/PET	freedom from in-field progression	100%	NR	clinical recurrence (RECIST/PERCIST)	53.3%	NR
Napieralska et al., 2016 [31]	CT scan/MR	dimensional increase of treated LN (RECIST)	78.5%	1y: 93% 2y: 70%	progression in uninvolved LN/other organs	56.3%	NR
Pasqualetti et al., 2016 [24]	BF: two consecutive PSA rises over 6 weeks- > choline PET	no recurrence in treated lesions	100%	NR	NR	NR	NR
Bouman-Wammes et al., 2017 [34]	BF: PSA increase ≥ 25% from baseline or increase of 2 ng/ml- > choline PET	no uptake/strongly diminished uptake in the high dose radiation volume	100%	NR	NR	NR	NR
Franzese et al., 2017 [32]	BF: PSA > PSA nadir + 2 ng/ml- > choline PET/TC scan	freedom from in-field progression	77% pp 63.2% pl	1y: 79.6% 2y: 74.9% 3y: 74.9%	NR	NR	1y: 55.2% 2y: 35.1%
Ingrosso et al., 2017 [33]	BF: PSA increase ≥ 20% from pre-SBRT value-> choline PET	freedom from in-field progression	98%	NR	NR	40% (60% PD: 40% LN out of field, 7.5% bone, 5% lymphatic spread, 5% prostate bed, 2.5% bone and liver)	NR
Jereczek-Fossa et al., 2017 [29]	BF: PSA increase > 10% from pre-SBRT value- > choline PET	freedom from in-field progression	90.3%	2y: 84%	Biochemical and/or clinical failure	35.1% (64.9% PD: 31.9% biochemical recurrence, 11.7% locoregional LN, 21% distant mets)	2y: 30%
Kneebone et al., 2018 [23]	BF: PSA > PSA nadir + 0.2 ng/ml- > PSMA-PET	freedom from in-field progression	100%	NR	biochemical and/or clinical failure	29.7% (70.3% PD: 13.5% biochemical recurrence,43.2% LN, 8.1% bone, 5.4% mixed)	NR
Siva et al., 2018 [37]	CT	local progression defined as increase of ≥ 20% in the largest tumor dimension with minimal absolute increase of 5 mm (RECIST)	NR	2y: 100%	NR	27.3% (11 evaluable pts with pelvic LN mets), (72.7% PD: 36.4% pelvic LN, 36.4% extra pelvic LN)	2y: 42%
Oehler et al., 2019 [35]	At discretion of the physician	in-field relapse defined as recurrence inside the PTV	97.2%	NR	recurrence outside the PTV	NR	NR
Ong et al., 2019 [36]	BF: PSA increase > 50% from pre-SBRT value, or 3 consecutive rises—> PSMA-PET	local progression defined as PSMA-avid disease progression within the 20% isodose line	95.2%	NR	PSMA-avid disease progression outside the SBRT treatment field	58.8%	NR

*BF* biochemical failure, *CT* computed tomography, *mo* months, *LN* lymph node(s), *mets* metastases, *MR* magnetic resonance, *PET* positron emission tomography, *pp* per patient, *pl* per lesion

*7/25 patients were treated with whole pelvis RT + SBRT

#### Progression-free survival

Five studies specified the site of treatment failure (i.e., out of field nodal progression, bone or visceral metastases, prostate bed recurrence) [23, 25, 32, 33, 37]. PFS was reported as crude rate or calculated with actuarial method or both in seven [23, 25, 28, 30, 31, 33, 36], one [32], and four [26, 27, 29, 37] studies, respectively. Actuarial rates were reported as 1-, 2-, 2.5-, and 3-year PFS in two [26, 32], four [26, 29, 32, 37], one [27], and one study [26], respectively (Table 3).

#### Other outcomes

BRes was reported in four studies [27, 29, 30, 35]: three of them [27, 29, 35] considered as complete biochemical response a PSA reduction > 50%, as minor response a 10–50% reduction and as stable disease a PSA modification between -10% and + 10%, while the forth study did not specify any threshold for BRes definition [30]. Biochemical relapse, defined as PSA increase after an initial (at least partial) PSA response, was reported in four studies [30, 32, 33, 35]. CRes was assessed in two studies [26–32], based on post-SBRT choline PET. In two studies [27–29], data on CRes were not available for all patients, and therefore were not considered in our analysis. OS and ADT-FS were reported in three [26, 31, 33] and four [29, 33, 35, 36] studies, respectively. Notably, ADT-FS was reported in four studies but referred to the whole patients' cohort [24, 25, 34, 37], and therefore not considered for the aim of this review. Toxicity was separately reported for patients with LN metastases in nine studies [25–33], while in four studies it was reported for the whole cohort of patients [23, 24, 34, 37], and therefore it was not included in the analysis. Moreover, toxicity was scored using the RTOG/EORTC scale in five studies [26–28, 31, 33] and with CTCAE criteria in three studies [25, 30, 32]. Details on secondary outcomes are reported in Table 4.

**Table 4:** Secondary outcomes

Author, year	Biochemical response	Biochemical relapse	clinical response	ADT-FS	OS
Jereczek-Fossa et al., 2009 [28]	NR	NR	NR	NR	NR
Casamassima et al., 2011 [26]	NR	NR	complete regression at 60-days PET: 56.5%	NR	1y: 92.0%, 2y: 92.0% 3y: 92.0%
Jereczek-Fossa et al., 2012 [27]	75.0% CR, 6.0% minor biochemical response, 13.0% stable PSA, 6.0% biochemical progression	NR	NR§	NR	NR
Decaestecker et al., 2014 [25]	NR	NR	NR	NR*	NR
Detti et al., 2015 [30]	CR 70.6%, biochemical progression 26.5%	Median time to biochemical recurrence 8.1 mo (considering only patients with recurrence-16/34: 6.9 mo, range 2.0–16.8 mo)	NR§	NR	NR
Napieralska et al., 2016 [31]	NR	NR	NR	NR	1y: 100% 2y: 67.0%
Pasqualetti et al., 2016 [24]	NR	NR	NR	NR*	NR
Bouman-Wammes et al., 2017 [34]	NR	NR	NR	NR*	NR
Franzese et al., 2017 [32]	NR	biochemical recurrence: 73.0%; mean time 15.3 mo (1.6–127.3)	metabolic response to post-SBRT PET: CR 44.7%, PR 38.0%, SD 7.9%, PD 7.9%	NR	NR
Ingrosso et al., 2017 [33]	NR	2-y bPFS 44.0%; median bPFS 24 mo; mean time to biochemical recurrence: 15.5 mo (1.2–48.9)	NR	ADT-FS 40.0% (21 evaluable pts); median ADT-FS 13.6 mo (2.1–37.1)	95.0% (crude)
Jereczek-Fossa et al., 2017 [29]	At 3 mo: 78.7% CR or stable PSA	NR	NR	median 7.2 mo (2.4–32.1); in 38.0% of pts ADT-FS > 12 mo	NR
Kneebone et al., 2018 [23]	NR	NR	NR	NR	NR
Siva et al., 2018 [37]	NR	NR	NR	NR*	NR
Oehler et al., 2019 [35]	52% CR, 16% minor biochemical response, 24% biochemical progression	Median time to biochemical recurrence 10.8 mo (small LN, ≤ 14 mm) vs 21.2 mo (large LN, > 14 mm)	NR	68.0% (crude)	NR
Ong et al., 2019 [36]	NR	NR	NR	1y ADT-FS: 70%°	NR

*ADT-FS* androgen deprivation therapy free survival, *bPFS* biochemical progression free survival, *CR* complete response, *LN* lymph nodes, *mo* months, *NR* not reported, *PD* progressive disease, *PET* positron emission tomography, *PR* partial response, *pts* patients, *SBRT* stereotactic body radiation therapy, *SD* stable disease, *y* year

*Data were reported for whole cohort only, and therefore excluded from the analysis

^§^Not available for all pts, and therefore excluded from the analysis

°Result referred to the whole cohort, included because 85% of patients met our inclusion criteria

### Main outcomes

#### Local control

When reported as crude percentage, LC was 100% in seven out of 13 studies [23–25, 27, 28, 30, 34] and ranged from 63.2 to 98.0% in the other six studies [29, 31–33, 35, 36]. Notably, four [23–25, 34] of the seven series with 100% LC were studies also including bone metastases and with separate outcomes for the different metastatic sites not explicitly reported. However, being overall LC rate 100% (Fig. 1), and we inferred that LC in LN metastases was 100% too, and therefore we chose to include these data in our report. All papers with actuarial evaluation of LC [26, 29, 31, 32, 37] reported the 2-year rates (range 70–100%, median: 84.0%) (Table 3). Notably, only in three series, some imaging examination was routinely performed during FU [26, 31, 37]; in the other studies, PET/CT or CT scan or MRI were performed only in case of biochemical failure (BF) [23–25, 27–30, 32–36]. Since the definition of BF varied between the studies, a misdetection of local recurrence associated with small increases of PSA cannot be excluded. For example, Jereczek-Fossa et al. [27, 29], Ingrosso et al. [33]and Ong et al. [36] considered as a threshold for imaging restaging a PSA increase from pre-SBRT value ≥ 10%, ≥ 20% and > 50%, respectively, while Oehler et al. [35]and Bouman-Wammes et al. [34] considered a threshold for restaging a PSA increase ≥ 25% or ≥ 2 ng/ml from pre-SBRT value. Kneebone et al. [23]and Jereczek-Fossa et al. [28] performed imaging exams in patients with PSA increase above the nadir > 0.2 ng/ml and 0.1 ng/ml, respectively. Other 4 studies did not specify any threshold for imaging restaging [24, 25, 30, 32].

#### Progression-free survival

PFS was reported as crude rate in 11 studies and ranged from 27.3% to 68.8% (median:42.9%) [23, 25–31, 33, 36, 37]. Actuarial PFS was reported in five studies and the median 2-year PFS was 38.6% (four studies, range 35.1–50.0%) [26, 29, 32, 37]. Only two studies reported 1-year PFS, with quite different results: Casamassima et al. [26] reported 80% 1-year PFS *versus* 55.2% reported by Franzese et al. [32]. Notably, in Casamassima et al. series seven out of 25 patients (28%) were treated with ENI associated with SIB to PET-positive LN. Finally, Jereczek-Fossa et al. reported 63.5% 2.5-year PFS. A similar result was reported by the same research group in 2017, with 67.1% of clinical PFS extrapolated from the presented data [29]. In fact, Jereczek-Fossa et al. reported crude 64.9% disease progression rate, that included half of patients (32.0%) with biochemical recurrence only, without other evidence of disease (and therefore not included in our PFS analysis) (Table 3).

#### Other outcomes

BRes rates after SBRT were evaluated in four studies [27, 29, 30, 35], with complete BRes ranging from 52.0% to 78.7% (median 63.5%). Biochemical relapse rates were reported in four studies [29, 32, 33, 35], with a median time to biochemical recurrence of 15.3 months (range 21.2–8.1 months) (Table 4). CRes, defined as regression of the treated LN at post-SBRT choline PET evaluation, was reported by Casamassima et al. [26]as 56.5% of “complete regression at 60 days-PET”, while Franzese et al. [32] reported 44.7% complete CRes and 38.0% partial CRes at post-SBRT choline PET/TC scan using the PERCIST/RECIST criteria. Three studies reported OS: Casamassima et al. [26] reported 92.0% 1-, 2- and 3-year OS, while Napieralska [31] et al. reported 100% and 67% 1- and 2-year OS rates, respectively. Ingrosso et al. [33] reported 95.0% crude OS (median FU: 23.8 months). Most of the series reported data on ADT-FS [24, 25, 29, 33–37]. However, in most of them [24, 25, 34, 37], this data was reported for the whole patients' cohort, without distinction between patients with LN or bone metastases. Therefore, these data were not considered in our report. Crude ADT-FS was 68.0% and 40.0% at the last FU in Oehler et al. [35] and Ingrosso et al. [33] series, respectively. Jereczek-Fossa et al. in 2017 [29] reported data on 94 patients, 60 of whom treated with SBRT without ADT; 36.0% of them started ADT during FU for disease progression, with a median ADT-FS of 7.2 months (2.4–32.1). Notably, in 38.0% of these patients, ADT-FS was > 12 months (Table 4). Ong et al. [36] reported 70.0% 1-year ADT-FS in the whole patients' cohort. However, since patients with bone metastases without LN metastases were only 3 out of 20, we decided to report this data in our analysis. Toxicity was reported in most studies and was usually mild. On 322 evaluable patients in terms of side effects out of 414 total patients included in our analysis, only two G3 acute toxicity (0.6%) [27, 37] and two G3 late toxicities (0.6%) [27, 33] were reported, without any > G3 toxicity (Table 5). Considering mild and moderate acute toxicity (G1-2) after nodal SBRT [25–33] none of the studies exceeded 20% (median 2.9%, range 0–19.2%). The highest G1-2 late toxicity rates were reported by Jereczek-Fossa et al. in 2012 (33.3%) [27] and 2009 (16.7%) [28]. In the other studies reporting G1-2 late toxicity rates [25, 30–33], the median value was 1.8% (range 0.5–3.0%).

**Table 5:** Acute and late toxicity

Author, year	Reported for whole cohort/ NM	Evaluated patients	Scale	Acute toxicity G1-2, n (%)	Late toxicity G1-2, n (%)	Acute toxicity G3, n (%)	Late toxicity G3, n (%)
Jereczek-Fossa et al., 2009 [28]	NM	6	RTOG/EORTC	0%	1 G2 (16.7%)	0%	0%
Casamassima et al., 2011 [26]	NM	NR	RTOG/EORTC	NO toxicity > G1	NR	0%	NR
Jereczek-Fossa et al., 2012 [27]	NM	12	RTOG/EORTC	0%	3 G1 (25%) 1 G2 (8.3%)	1 (8.3%)	1 (8.3%)
Decaestecker et al., 2014 [25]	NM	27	CTCAE v.3	3 G1 (11.1%), 2 G2 (7.4%)	1 G2 (3.7%)	0%	0%
Detti et al., 2015 [30]	NM	30	CTCAE v.4	1 G2 (3.3%)	1 G1 (NR) §	0%	0%
Napieralska et al., 2016 [31]	NM	NR	RTOG/EORTC	0%	3 G1 (NR) *	0%	0%
Pasqualetti et al., 2016 [24]	Whole cohort	NR	CTCAE v.4	NO toxicity > G1	NO toxicity > G1	0%	0%
Bouman-Wammes et al., 2017 [34]	Whole cohort	43	NR	2 G1 (4.6%) 2 G2 (4.6%)	0%	0%	0%
Franzese et al., 2017 [32]	NM	26	CTCAE v.4	5 G1 (19.2%)	0%	0%	0%
Ingrosso et al., 2017 [33]	NM	40	RTOG/EORTC	1 (2.5%)	0%	0%	1 (2.5%)
Jereczek-Fossa et al., 2017 [29]	NM	94	NR	7 G1 (7.4%) 1 G2 (1.1%)	2 G1 (2.1%) 3 G2 (3.2%)	0%	0%
Kneebone et al., 2018 [23]	Whole cohort	45	CTCAE v.4	4 G1 (8.9%) 1 G2 (2.2%)	5 G1 (11.1%)	0%	0%
Siva et al., 2018 [37]	Whole cohort	33	CTCAE v.4	16 G1 (48.5%) 5 G2 (15.1%)	NR	1 (3.0%)	NR
Oehler et al., 2019 [35]	NR	NR	NR	NR	NR	NR	NR
Ong et al., 2019 [36]	NR	NR	NR	NR	NR	NR	NR

*CTCAE v.3/4* Common Terminology Criteria for Adverse Events version 3/4, *G* grade, *n* number, *NM* nodal metastases, *NR* not reported, *RTOG/EORTC* Radiation Therapy Oncology Group/European Organization for Research and Treatment of Cancer

*Between patients with at least 3 months of FU

^§^Between patients with at least 6 months of FU

## Discussion

The interest in MDT for oligometastatic PCa is growing, but strong evidence on patients’ selection and treatment modalities is still lacking [8]. Nodal metachronous oligometastases seem to identify an early step in PCa progression, and thus they should be analyzed separately from bone and visceral metastases [38]. Moreover, the possibility of identifying early metachronous oligometastatic PCa using specific radiotracer (choline, PSMA) provides the chance to perform effective MDT. However, some authors believe that a consequential risk of using these imaging techniques is to mainly identify patients with indolent disease [3]. Therefore, patients’ selection is still a critical issue in this scenario, and our search aimed to select the relatively homogeneous population of nodal metachronous oligorecurrence from PCa treated with SBRT to summarize the currently available knowledge.

For this reason, papers where clinical results of SBRT for LN metastases where not clearly reported [39–41] were excluded, as well as studies with partially duplicated data whenever it was impossible to obtain information only for the originally reported ones [23–25, 27, 34, 36, 37]. Furthermore, techniques different from SBRT and oligometastatic PCa involving bone and viscera were considered exclusion criteria. Despite these efforts, the main limitation of our study is the non-negligible heterogeneity in patients populations, partially explained by the retrospective design of most analyzed studies. Particularly, the more relevant sources of variability were hormonal status, maximum number of metastases per patients, and combination of SBRT with ENI and/or ADT.

In fact, several studies included patients with mixed hormonal status [23, 25, 37] or did not report this characteristic [24, 26–33, 36]. Only Oehler et al. [35]and Bouman-Wammes et al. [34] included in their studies a homogeneous population of hormone-naive and hormone-sensitive patients, respectively. Moreover, the number of patients treated on a single lesion ranged among half of subjects [31] to over 90% of patients [37]. Treatments combined with SBRT were another source of variability. For example, Casamassima et al. [26]and Napieralska et al. [31] included in their case series28% and 11.1% of patients to whom SBRT was administered as a SIB during ENI, respectively. The authors of these studies reported that this treatment modality seemed to improve clinical outcomes. Furthermore, the combination of ADT with SBRT was not allowed in 5 studies [23, 25, 34–36], while the percentage of patients receiving ADT ranged from 33.3% to 100% in seven reports [27–33]. Obviously, this variability could have influenced the outcome in terms of PFS. Moreover, considering the retrospective design of most studies, almost half of the series included 1–2 patients with metachronous oligometastases to both LNs and bones [23, 24, 31, 34, 36, 37]. Finally, Napieralska et al. [31] included in their series two patients with synchronous oligometastatic disease, who received SBRT as a component of the primary treatment. However, we choose to include these papers in our analysis given the small percentage of these cases (2.9–11.7%). Nevertheless, though these numbers are small, we cannot exclude an effect on overall outcomes, particularly in terms of PFS.

Another limit of our analysis is that we included four studies [23–25, 34] reporting 100% LC in patients treated for LN and bone metastases, for which separate outcomes were not reported, because we can infer that LC for LN metastases was 100% as well. This choice may have led to a selection bias because similar studies (not reporting separated results in PCa patients with LN and bone oligometastases) with < 100% rates of LC were excluded being impossible to ascribe the LN metastases specific LC rate.

Despite these limits, we found that LC was high in all analyzed studies, even if only a minority of them reported a clear definition of “in-field recurrence” [25, 31, 36, 37]. More generally, SBRT seems effective in “neutralizing” the target lesion, usually in a lasting way. In fact, in series reporting both 2- and 3-year LC rates [26, 32] the result remained stable over time. However, Napieralska et al. [31]and Franzese et al. [32] reported the lowest LC rates (crude LC rate of 78.5% and 63.2%, respectively). The former authors stated that their priority was not to exceed the OaRs constraints and that, in some cases, the minimum dose to the PTV was < 95%. Moreover, the authors stated that both total dose and dose per fraction increased during the study period, as long as more evidence on SBRT safety became available. Notably, they defined LC based on CT/MRI instead of PET, used in most studies. The combination of all these features could explain the reported LC rates. Similarly, Franzese et al. [32] reported that alternative schedules were adopted when OaRs constraints were not met. Again, this might probably explain the low (74.9% 2- and 3-year) LC rate, even though a clear definition of LC was lacking. Beyond these two studies, all other series reported LC rate ranging between 90.3% and 100%. Therefore, our analysis confirms the efficacy of SBRT in providing high LC rates in nodal metastases, even in the setting of PCa oligorecurrences. Finally, in their recent review and meta-analysis [42], Yan et al. reported data on SBRT as MDT in oligometastatic PCa patients, with both LN and bone metastases. The analysis showed 97% overall LC and 39% 2-year PFS, which are consistent with the findings of the present study.

Despite the satisfactory results in terms of LC, PFS rates were low and steeply decreasing over time in most reports. In fact, in Casamassima et al. series [26] the PFS was 80% at 1 year but 50% and 17% at 2 and 3 years, respectively. Moreover, in Franzese et al. series [32], the PFS rate fell from 55 to 35% between the first and the second year after SBRT. The worst result was reported by Jereczek-Fossa et al. [29], who recorded 30% 2-year PFS rates. However, it should be noted that PFS was defined as both clinical and biochemical recurrence and that half of recorded events were isolated biochemical recurrence (32.0% out of 64.9% disease progressions). Similarly, Kneebone et al. [23] reported 29.7% crude PFS including 13.5% isolated biochemical recurrence. The better result was reported in another study by Jereczek-Fossa et al. (crude PFS: 68.8%, 30 months-PFS: 63.5%) [27]. Interestingly, in all Jereczek-Fossa’s studies included in our analysis [27–29] an exclusion criterion was an interval between primary treatment and oligorecurrence > 24 months. Therefore, the positive results recorded in these series could derive from the enrollment of patients with less aggressive neoplasms. Phillips et al. recently published the results of the ORIOLE trial [43] on oligometastatic PCa. The authors reported 81% and 39% 6-months PFS in the SBRT and observation arms, respectively. Moreover, with 18.8 months median FU, the median PFS was not reached and 5.8 months in the SBRT and in the observation arm, respectively. This is consistent with the results of the SABR-COMET trial [44], where patients who received standard-of-care treatments combined with SBRT showed 25% absolute 5-year survival benefit compared to the standard-of-care therapy alone arm.

Other studies reported data on different MDT strategies in the same setting. In a recent review, Ploussard et al*.* [45] reported the results of salvage LN dissection (sLND), with complete BRes and 2-year PFS rates ranging from 13 to 80% and from 23 to 64%, respectively. However, G3 postoperative complications were reported in most series, with an incidence of up to 20% (mainly lymphocele drainage, ureteral stenting, sepsis, pulmonary embolism). Furthermore, De Bruycker et al. [46] compared sLND and ENI as salvage treatment approach analyzing the anatomical distribution of nodal oligorecurrences. The authors reported better coverage with ENI or super extended sLND compared to limited or standard sLND. Moreover, some papers reported comparisons between ENI and SBRT (or other MDTs). In fact, De Bleser et al. [47] found that ENI (with or without SIB) may reduce recurrences compared with SBRT alone in solitary LN metastases, being associated with a significantly lower nodal recurrences rate (20% *versus* 42%) and with prolonged metastasis-free survival (HR: 0.5, 95% CI 0.30–0.85, p = 0.009). However, the authors also reported higher toxicity rates after ENI, compared to SBRT (late toxicity: 18% *versus* 6%, G3-4 late toxicity: 2.5% *versus* 0%, respectively). Furthermore, Lépinoy et al. [48] reported 88% and 55% 3-year PFS after ENI and MDT to the involved LNs, respectively. Finally, Jethwa et al. [49] reported encouraging results after the combination of ENI with SIB and ADT with 79% 2-year biochemical PFS and 98% and 47% 4-year OS and biochemical PFS, respectively. The rate of in field recurrences was 1% and 6% at 2 and 4 years, respectively, and the incidence of out-of-field recurrence was 6% and 24% at 2 and 4 years, respectively.

Taken together, these data suggest that sLND should not be considered a standard of care for nodal metachronous oligometastatic PCa but rather an investigational treatment [50]. Conversely, ENI should be evaluated as a part of multimodal approach including SBRT-boost on the involved LNs. In fact, a recent DEGRO PCa expert panel [51] recommended to treat pelvic only oligorecurrent nodal metastases from PCa with ENI *plus* a boost to the involved LNs, and to consider SBRT alone in nodal extra pelvic oligorecurrences. In both cases, systemic therapies should be prescribed according to guidelines. However, in some low-risk situations (i.e., PSA doubling time > 10 months and relapse free interval from initial curative treatment > 2 years) an upfront local treatment could be considered. In fact, another goal of some studies on SBRT in this setting was to delay the onset of ADT. In two series the rate of oligorecurrent patients free from ADT after SBRT was 40% and 68% [33, 35]. Furthermore, Ong et al. [36] reported 70% 1-year ADT-FS while Ingrosso et al. and Jereczek-Fossa et al. [29, 33] reported 13.6- and 7.2-months median ADT-FS, respectively. Higher figures were recorded in the STOMP trial [12] where median ADT-FS was 21 months and 14 months in the MDT and surveillance arms, respectively. Moreover, the updated results of the trial [52] showed 34% and 8% 5-year ADT-free survival in the MTD and surveillance arms, respectively. The difference between the STOMP trial and the series included in our analysis could result from the different ways of managing hormone therapy after MDT. In fact, in the STOMP trial the use of ADT was reserved for patients with progression in more than three metastases, symptomatic progression, or local progression of metastatic sites compared to the pretreatment assessment, while only an increased PSA was not a sufficient criterion. In contrast, in the series included in our analysis, the management of patients after SBRT was left to the discretion of the treating radiation oncologists. [39]

Our analysis confirms that SBRT is a well-tolerated treatment option, with only two G3 acute toxicity [27, 37] and two G3 late toxicities [27–33] in more than 300 evaluable patients. Moreover, mild and moderate acute toxicity never exceeded 20%. However, Siva et al. [37], who reported the results of a phase II trial not included in our analysis due to the inclusion of both LN and bone metastases, reported 63.6% acute G 1–2 toxicity rates. This difference may suggest that toxicity rates collected in a prospective setting are higher compared to retrospectively collected data, especially when considering mild to moderate toxicity.

## Conclusion

Our results strongly suggest that SBRT of oligometastatic nodal metachronous PCa is well tolerated and provides satisfactory and long-lasting LC, while PFS rates show a progressive and rather rapid reduction over time. Furthermore, SBRT would allow for a delay in ADT onset, with a potential positive impact on quality of life. Unfortunately, only few data on OS are available in the analyzed series. Although PFS was sometimes proposed as a surrogate endpoint for OS [53], this approach would not seem needful in the metastatic setting, where the short FU period allows for direct assessment of OS.

The use of ADT is still a topic of debate. In fact, SBRT was used both to delay the ADT onset and to improve the ADT results through local treatment intensification. [25, 53, 54]. Carrasquilla et al. [55] have recently proposed the combination of intermittent ADT *plus* MDT based on SBRT delivered with an “involved field” strategy including two dose levels: GTV and high-risk CTV (GTV *plus* the adjacent LN basins). This compromise solution, through avoiding both standard ENI and prolonged and ongoing ADT, could allow for a reduction in adverse events and a consequent improvement in quality of life.

The heterogeneity of the analyzed series reflects the still open questions on the selection of patients to be treated with SBRT alone with the aim to delay the ADT start. Hormone-naïve or -sensitive patients, with 1–2 regional involved LNs, with time interval between primary treatment and oligorecurrence ≥ 24 months, and with “slow growing” PSA are theoretically the best candidates. In fact, in these subjects the risk of misdiagnosing disseminated micrometastatic disease as oligorecurrent PCa would be minimized. [56] However, an argument against this hypothesis is that these patients could be the ones with latent metastatic PCa, which was simply not detected in the past due to less sensitive tracer (given the low metabolic uptake of these lesions) and which have a very good prognosis even without any intervention. Furthermore, it could be hypothesized that even selected tumors with short PSA doubling time could be managed with MDT, given the possibility of repeating the latter until widespread metastatic diffusion. Therefore, the aim of SBRT in this setting could be to make chronic oligometastases from PCa. [25, 39]. A frequent observation supporting repeated MDTs is that patients treated on LN metastases tend to further relapse in other LNs [23, 24, 34, 35, 37]. Unfortunately, most analyzed papers did not report details on the relapse sites after SBRT. Therefore, it remains unclear whether this pattern of recurrence is related to a particular subset of oligometastatic disease (with predominant lymphatic *versus* hematogenous spread) or if it is simply related to inadequate regional control. However, the tendency of metachronous oligometastatic PCa to relapse again as oligometastatic disease was confirmed also by Soldatov’s et al. [57] and Ost’s et al. [38] recent studies. This evidence seems to suggest that MDT could play a role, especially as a part of multimodal systemic and locoregional approach, even in higher risk patients, as proposed also by Ahmed et al. [58] in a recent review.

In conclusion, until the results of clinical trials (OLIGOPELVIS-2, STORM) will be available, several questions on SBRT of nodal metachronous oligometastatic PCa will remain unanswered. In particular, data is needed on optimal combination of SBRT with ADT (and other systemic therapies) and with ENI, as well as a clear definition of patients suitable for a less aggressive approach or for an intensive multimodal treatment including SBRT. Finally, to provide clinically meaningful answers to these open questions will require reliable data on OS and cancer specific survival.

## Supplementary Information

Below is the link to the electronic supplementary material.

Supplementary file1 (DOCX 12 KB)

Supplementary file2 (DOCX 27 KB)

## Data Availability

Data supporting reported results can be found at Radiotherapy Unit of the IRCCS Azienda Ospedaliero-Universitaria di Bologna.
