# Supplementary material for: Stereotactic radiotherapy of nodal oligometastases from prostate cancer: a prisma-compliant systematic review
Source: Clin Exp Metastasis. 2022 Aug 18;39(6):845–63. doi: 10.1007/s10585-022-10183-6 (PMC9637632; doi:10.1007/s10585-022-10183-6)
Supplement: Supplementary file 1 — Supplementary file1 (DOCX 12 KB) [file 10585_2022_10183_MOESM1_ESM.docx]

# **Appendix 1**: Search strategy

**Medline (PubMed)**

(“lymph node” OR “LN” OR “ lymph nodal” OR “nodal metastasis” OR “nodal metastases” OR “lymph node” OR “lymph nodes”) AND (metastasis OR metastases OR metastatic OR neoplasm OR neoplasms OR cancer OR cancers OR carcinoma OR carcinomas OR adenocarcinoma OR adenocarcinomas OR tumor OR tumors OR tumor OR tumors) AND (radiosurgery OR “stereotactic body radiotherapy” OR “stereotactic body radiation therapy” OR “stereotactic body radiosurgery” OR “stereotactic radiotherapy” OR “stereotactic radiation therapy” OR “stereotactic radiosurgery” OR stereotaxis OR stereotaxic OR sbrt OR sbrs OR sabr OR “stereotactic ablative”) in “Title/Abstract”

**Scopus**

(“lymph node” OR “LN” OR “ lymph nodal” OR “nodal metastasis” OR “nodal metastases” OR “lymph node” OR “lymph nodes”) AND (metastasis OR metastases OR metastatic OR neoplasm OR neoplasms OR cancer OR cancers OR carcinoma OR carcinomas OR adenocarcinoma OR adenocarcinomas OR tumor OR tumors OR tumor OR tumors) AND (radiosurgery OR “stereotactic body radiotherapy” OR “stereotactic body radiation therapy” OR “stereotactic body radiosurgery” OR “stereotactic radiotherapy” OR “stereotactic radiation therapy” OR “stereotactic radiosurgery” OR stereotaxis OR stereotaxic OR sbrt OR sbrs OR sabr OR “stereotactic ablative”) in “ArticleTitle” and “Abstract”

**Cochrane**

(“lymph node” OR “LN” OR “ lymph nodal” OR “nodal metastasis” OR “nodal metastases” OR “lymph node” OR “lymph nodes”) AND (metastasis OR metastases OR metastatic OR neoplasm OR neoplasms OR cancer OR cancers OR carcinoma OR carcinomas OR adenocarcinoma OR adenocarcinomas OR tumor OR tumors OR tumor OR tumors) AND (radiosurgery OR “stereotactic body radiotherapy” OR “stereotactic body radiation therapy” OR “stereotactic body radiosurgery” OR “stereotactic radiotherapy” OR “stereotactic radiation therapy” OR “stereotactic radiosurgery” OR stereotaxis OR stereotaxic OR sbrt OR sbrs OR sabr OR “stereotactic ablative”) in “Title Abstract Keyword”
