# Supplementary material for: Stereotactic radiotherapy of nodal oligometastases from prostate cancer: a prisma-compliant systematic review
Source: Clin Exp Metastasis. 2022 Aug 18;39(6):845–63. doi: 10.1007/s10585-022-10183-6 (PMC9637632; doi:10.1007/s10585-022-10183-6)
Supplement: Supplementary file 2 — Supplementary file2 (DOCX 27 KB) [file 10585_2022_10183_MOESM2_ESM.docx]

**Appendix 2: Papers excluded after full text examination**

Study protocol

1. Berghen C, Joniau S, Rans K, Devos G, Poels K, Slabbaert K, Dumez H, Albersen M, Goffin K, Haustermans K, De Meerleer G. Metastasis-directed therapy in castration-refractory prostate cancer (MEDCARE): a non-randomized phase 2 trial. BMC Cancer. 2020 May 24;20(1):457. doi: 10.1186/s12885-020-06853-x. PMID: 32448171; PMCID: PMC7245754.
2. De Bruycker A, Spiessens A, Dirix P, Koutsouvelis N, Semac I, Liefhooghe N, Gomez-Iturriaga A, Everaerts W, Otte F, Papachristofilou A, Scorsetti M, Shelan M, Siva S, Ameye F, Guckenberger M, Heikkilä R, Putora PM, Zapatero A, Conde-Moreno A, Couñago F, Vanhoutte F, Goetghebeur E, Reynders D, Zilli T, Ost P. PEACE V - Salvage Treatment of OligoRecurrent nodal prostate cancer Metastases (STORM): a study protocol for a randomized controlled phase II trial. BMC Cancer. 2020 May 12;20(1):406. doi: 10.1186/s12885-020-06911-4. PMID: 32398040; PMCID: PMC7216526.
3. Decaestecker K, De Meerleer G, Ameye F, Fonteyne V, Lambert B, Joniau S, Delrue L, Billiet I, Duthoy W, Junius S, Huysse W, Lumen N, Ost P. Surveillance or metastasis-directed Therapy for OligoMetastatic Prostate cancer recurrence (STOMP): study protocol for a randomized phase II trial. BMC Cancer. 2014 Sep 15;14:671. doi: 10.1186/1471-2407-14-671. PMID: 25223986; PMCID: PMC4175227.

Imaging study

1. Farolfi A, Ceci F, Castellucci P, Graziani T, Siepe G, Lambertini A, Schiavina R, Lodi F, Morganti AG, Fanti S. ^68^Ga-PSMA-11 PET/CT in prostate cancer patients with biochemical recurrence after radical prostatectomy and PSA <0.5 ng/ml. Efficacy and impact on treatment strategy. Eur J Nucl Med Mol Imaging. 2019 Jan;46(1):11-19. doi: 10.1007/s00259-018-4066-4. Epub 2018 Jun 15. PMID: 29905907.
2. Kishan AU, Tyran M, Weng J, Upadhyaya S, Lamb J, Steinberg M, King C, Cao M. Stereotactic body radiotherapy to the prostate and pelvic lymph nodes: A detailed dosimetric analysis of a phase II prospective trial. Br J Radiol. 2019 Jul;92(1099):20181001. doi: 10.1259/bjr.20181001. Epub 2019 Apr 3. PMID: 30912957; PMCID: PMC6636273.
3. Winkel D, Bol GH, Werensteijn-Honingh AM, Intven MPW, Eppinga WSC, Hes J, Snoeren LMW, Sikkes GG, Gadellaa-van Hooijdonk CGM, Raaymakers BW, Jürgenliemk-Schulz IM, Kroon PS. Target coverage and dose criteria based evaluation of the first clinical 1.5T MR-linac SBRT treatments of lymph node oligometastases compared with conventional CBCT-linac treatment. Radiother Oncol. 2020 May;146:118-125. doi: 10.1016/j.radonc.2020.02.011. Epub 2020 Mar 6. PMID: 32146257.

Lesions considered/treatments delivered don’t fit inclusion criteria (NO prostate cancer, NO LN metastases, NO SBRT, NO LC/PFS)

1. Alsuhaibani A, Elashwah A, Alkafi A, Constantinescus C, ALzorkany F. Stereotactic Body Radiation Therapy (SBRT) Using CyberKnife in Oligometastatic Cancer Patients; Retrospective Evaluation, Single Institution Experience. J Gastrointest Cancer. 2019 Dec;50(4):879-887. doi: 10.1007/s12029-018-0170-8. PMID: 30291546. (no prostate)
2. Ahmed KA, Barney BM, Davis BJ, Park SS, Kwon ED, Olivier KR. Stereotactic body radiation therapy in the treatment of oligometastatic prostate cancer. Front Oncol. 2013 Jan 22;2:215. doi: 10.3389/fonc.2012.00215. PMID: 23346551; PMCID: PMC3551203. (<10 LN)
3. Corvò R, Lamanna G, Vagge S, Belgioia L, Bosetti D, Aloi D, Timon G, Bacigalupo A. Once-weekly stereotactic radiotherapy for patients with oligometastases: compliance and preliminary efficacy. Tumori. 2013 Mar-Apr;99(2):159-63. doi: 10.1700/1283.14186. PMID: 23748808. (no prostate)
4. Franceschini D, Bianciardi F, Mazzola R, De Rose F, Gentile P, Alongi F, Scorsetti M. Can thoracic nodes oligometastases be safely treated with image guided hypofractionated radiation therapy? Br J Radiol. 2019 Nov;92(1103):20181026. doi: 10.1259/bjr.20181026. Epub 2019 Sep 17. PMID: 31529985; PMCID: PMC6849678. (no prostate)
5. Ito M, Kodaira T, Koide Y, Okuda T, Mizumatsu S, Oshima Y, Takeuchi A, Mori T, Abe S, Asai A, Suzuki K. Role of high-dose salvage radiotherapy for oligometastases of the localised abdominal/pelvic lymph nodes: a retrospective study. BMC Cancer. 2020 Jun 9;20(1):540. doi: 10.1186/s12885-020-07033-7. PMID: 32517673; PMCID: PMC7285737. (no prostate)
6. Muldermans JL, Romak LB, Kwon ED, Park SS, Olivier KR. Stereotactic Body Radiation Therapy for Oligometastatic Prostate Cancer. Int J Radiat Oncol Biol Phys. 2016 Jun 1;95(2):696-702. doi: 10.1016/j.ijrobp.2016.01.032. Epub 2016 Jan 29. PMID: 27131082; PMCID: PMC5154616. (< 10 LN)
7. Palma DA, Olson R, Harrow S, Gaede S, Louie AV, Haasbeek C, Mulroy L, Lock M, Rodrigues GB, Yaremko BP, Schellenberg D, Ahmad B, Senthi S, Swaminath A, Kopek N, Liu M, Moore K, Currie S, Schlijper R, Bauman GS, Laba J, Qu XM, Warner A, Senan S. Stereotactic Ablative Radiotherapy for the Comprehensive Treatment of Oligometastatic Cancers: Long-Term Results of the SABR-COMET Phase II Randomized Trial. J Clin Oncol. 2020 Sep 1;38(25):2830-2838. doi: 10.1200/JCO.20.00818. Epub 2020 Jun 2. PMID: 32484754; PMCID: PMC7460150. (no LN)
8. Salama JK, Hasselle MD, Chmura SJ, Malik R, Mehta N, Yenice KM, Villaflor VM, Stadler WM, Hoffman PC, Cohen EE, Connell PP, Haraf DJ, Vokes EE, Hellman S, Weichselbaum RR. Stereotactic body radiotherapy for multisite extracranial oligometastases: final report of a dose escalation trial in patients with 1 to 5 sites of metastatic disease. Cancer. 2012 Jun 1;118(11):2962-70. doi: 10.1002/cncr.26611. Epub 2011 Oct 21. PMID: 22020702. (no prostate)
9. Tran S, Jorcano S, Falco T, Lamanna G, Miralbell R, Zilli T. Oligorecurrent Nodal Prostate Cancer: Long-term Results of an Elective Nodal Irradiation Approach. Am J Clin Oncol. 2018 Oct;41(10):960-962. doi: 10.1097/COC.0000000000000419. PMID: 29315174. (no SBRT)
10. Loi M, Di Cataldo V, Francolini G, Bonomo P, Masi L, Simontacchi G, Detti B, Greto D, Desideri I, Livi L. Single-Fraction Stereotactic Body Radiotherapy for Oligometastatic Lymph Node Relapse in Prostate Cancer. Oncol Res Treat. 2018;41(11):703-705. doi: 10.1159/000491605. Epub 2018 Oct 13. PMID: 30317236. (no LC/PFS)

Duplicated data

1. Alongi F, Fogliata A, Clerici E, Navarria P, Tozzi A, Comito T, Ascolese AM, Clivio A, Lobefalo F, Reggiori G, Cozzi L, Mancosu P, Tomatis S, Scorsetti M. Volumetric modulated arc therapy with flattening filter free beams for isolated abdominal/pelvic lymph nodes: report of dosimetric and early clinical results in oligometastatic patients. Radiat Oncol. 2012 Dec 5;7:204. doi: 10.1186/1748-717X-7-204. PMID: 23216821; PMCID: PMC3551769.
2. Cysouw M, Bouman-Wammes E, Hoekstra O, van den Eertwegh A, Piet M, van Moorselaar J, Boellaard R, Dahele M, Oprea-Lager D. Prognostic Value of [^18^F]-Fluoromethylcholine Positron Emission Tomography/Computed Tomography Before Stereotactic Body Radiation Therapy for Oligometastatic Prostate Cancer. Int J Radiat Oncol Biol Phys. 2018 Jun 1;101(2):406-410. doi: 10.1016/j.ijrobp.2018.02.005. Epub 2018 Feb 13. PMID: 29559285.
3. De Bleser E, Jereczek-Fossa BA, Pasquier D, Zilli T, Van As N, Siva S, Fodor A, Dirix P, Gomez-Iturriaga A, Trippa F, Detti B, Ingrosso G, Triggiani L, Bruni A, Alongi F, Reynders D, De Meerleer G, Surgo A, Loukili K, Miralbell R, Silva P, Chander S, Di Muzio NG, Maranzano E, Francolini G, Lancia A, Tree A, Deantoni CL, Ponti E, Marvaso G, Goetghebeur E, Ost P. Metastasis-directed Therapy in Treating Nodal Oligorecurrent Prostate Cancer: A Multi-institutional Analysis Comparing the Outcome and Toxicity of Stereotactic Body Radiotherapy and Elective Nodal Radiotherapy. Eur Urol. 2019 Dec;76(6):732-739. doi: 10.1016/j.eururo.2019.07.009. Epub 2019 Jul 20. PMID: 31331782.
4. Jereczek-Fossa BA, Piperno G, Ronchi S, Catalano G, Fodor C, Cambria R, Fossati Ing P, Gherardi F, Alterio D, Zerini D, Garibaldi C, Baroni G, De Cobelli O, Orecchia R. Linac-based stereotactic body radiotherapy for oligometastatic patients with single abdominal lymph node recurrent cancer. Am J Clin Oncol. 2014 Jun;37(3):227-33. doi: 10.1097/COC.0b013e3182610878. PMID: 22992626.
5. Nicosia L, Franzese C, Mazzola R, Franceschini D, Rigo M, D'agostino G, Corradini S, Alongi F, Scorsetti M. Recurrence pattern of stereotactic body radiotherapy in oligometastatic prostate cancer: a multi-institutional analysis. Strahlenther Onkol. 2020 Mar;196(3):213-221. English. doi: 10.1007/s00066-019-01523-9. Epub 2019 Sep 26. PMID: 31559480.
6. Ost P, Jereczek-Fossa BA, Van As N, Zilli T, Tree A, Henderson D, Orecchia R, Casamassima F, Surgo A, Miralbell R, De Meerleer G. Pattern of Progression after Stereotactic Body Radiotherapy for Oligometastatic Prostate Cancer Nodal Recurrences. Clin Oncol (R Coll Radiol). 2016 Sep;28(9):e115-20. doi: 10.1016/j.clon.2016.04.040. Epub 2016 Apr 28. PMID: 27133946.
7. Ponti E, Ingrosso G, Carosi A, Di Murro L, Lancia A, Pietrasanta F, Santoni R. Salvage Stereotactic Body Radiotherapy for Patients With Prostate Cancer With Isolated Lymph Node Metastasis: A Single-Center Experience. Clin Genitourin Cancer. 2015 Aug;13(4):e279-e284. doi: 10.1016/j.clgc.2014.12.014. Epub 2014 Dec 30. PMID: 25604915.
8. Triggiani L, Mazzola R, Magrini SM, Ingrosso G, Borghetti P, Trippa F, Lancia A, Detti B, Francolini G, Matrone F, Bortolus R, Fanetti G, Maranzano E, Pasqualetti F, Paiar F, Bonù ML, Magli A, Bruni A, Mazzeo E, Franzese C, Scorsetti M, Alongi F, Jereczek-Fossa BA, Ost P, Buglione M. Metastasis-directed stereotactic radiotherapy for oligoprogressive castration-resistant prostate cancer: a multicenter study. World J Urol. 2019 Dec;37(12):2631-2637. doi: 10.1007/s00345-019-02717-7. Epub 2019 Mar 11. PMID: 30859273.

Not separated results

1. Berkovic P, De Meerleer G, Delrue L, Lambert B, Fonteyne V, Lumen N, Decaestecker K, Villeirs G, Vuye P, Ost P. Salvage stereotactic body radiotherapy for patients with limited prostate cancer metastases: deferring androgen deprivation therapy. Clin Genitourin Cancer. 2013 Mar;11(1):27-32. doi: 10.1016/j.clgc.2012.08.003. Epub 2012 Sep 24. PMID: 23010414.
2. Bowden P, See AW, Frydenberg M, Haxhimolla H, Costello AJ, Moon D, Ruljancich P, Grummet J, Crosthwaite A, Pranavan G, Peters JS, So K, Gwini SM, McKenzie DP, Nolan S, Smyth LML, Everitt C. Fractionated stereotactic body radiotherapy for up to five prostate cancer oligometastases: Interim outcomes of a prospective clinical trial. Int J Cancer. 2020 Jan 1;146(1):161-168. doi: 10.1002/ijc.32509. Epub 2019 Jun 28. PMID: 31199504.
3. Deek MP, Yu C, Phillips R, Song DY, Deville C, Greco S, DeWeese TL, Antonarakis ES, Markowski M, Paller C, Denmeade S, Carducci M, Walsh PC, Pienta KJ, Eisenberger M, Tran PT. Radiation Therapy in the Definitive Management of Oligometastatic Prostate Cancer: The Johns Hopkins Experience. Int J Radiat Oncol Biol Phys. 2019 Dec 1;105(5):948-956. doi: 10.1016/j.ijrobp.2019.08.008. Epub 2019 Aug 13. PMID: 31419509; PMCID: PMC7050213.
4. Deek MP, Taparra K, Phillips R, Velho PI, Gao RW, Deville C, Song DY, Greco S, Carducci M, Eisenberger M, DeWeese TL, Denmeade S, Pienta K, Paller CJ, Antonarakis ES, Olivier KR, Park SS, Tran PT, Stish BJ. Metastasis-directed Therapy Prolongs Efficacy of Systemic Therapy and Improves Clinical Outcomes in Oligoprogressive Castration-resistant Prostate Cancer. Eur Urol Oncol. 2021 Jun;4(3):447-455. doi: 10.1016/j.euo.2020.05.004. Epub 2020 Jun 11. PMID: 32536574; PMCID: PMC7788526.
5. Deodato F, Ferro M, Cilla S, Ianiro A, Buwenge M, Re A, Sallustio G, Valentini V, Morganti AG, Macchia G. Stereobody radiotherapy for nodal recurrences in oligometastatic patients: a pooled analysis from two phase I clinical trials. Clin Exp Metastasis. 2020 Aug;37(4):519-529. doi: 10.1007/s10585-020-10039-x. Epub 2020 Jun 3. PMID: 32495238.
6. Franceschini D, De Rose F, Franzese C, Comito T, Di Brina L, Radicioni G, Evangelista A, D'Agostino GR, Navarria P, Scorsetti M. Predictive Factors for Response and Survival in a Cohort of Oligometastatic Patients Treated With Stereotactic Body Radiation Therapy. Int J Radiat Oncol Biol Phys. 2019 May 1;104(1):111-121. doi: 10.1016/j.ijrobp.2018.12.049. Epub 2019 Jan 8. PMID: 30630030.
7. Franzese C, Zucali PA, Di Brina L, D'Agostino G, Navarria P, Franceschini D, Santoro A, Scorsetti M. The efficacy of Stereotactic body radiation therapy and the impact of systemic treatments in oligometastatic patients from prostate cancer. Cancer Med. 2018 Sep;7(9):4379-4386. doi: 10.1002/cam4.1707. Epub 2018 Aug 2. PMID: 30073758; PMCID: PMC6144154.
8. Franzese C, Comito T, Tripoli A, Franceschini D, Clerici E, Navarria P, Badalamenti M, D'agostino G, Loi M, Mancosu P, Reggiori G, Tomatis S, Scorsetti M. Phase II trial of high dose stereotactic body radiation therapy for lymph node oligometastases. Clin Exp Metastasis. 2020 Oct;37(5):565-573. doi: 10.1007/s10585-020-10047-x. Epub 2020 Jun 15. PMID: 32556682.
9. Franzese C, Badalamenti M, Comito T, Franceschini D, Clerici E, Navarria P, Loi M, D'agostino G, Baldaccini D, Chiola I, Reggiori G, Mancosu P, Tomatis S, Scorsetti M. Assessing the role of Stereotactic Body Radiation Therapy in a large cohort of patients with lymph node oligometastases: Does it affect systemic treatment's intensification? Radiother Oncol. 2020 Sep;150:184-190. doi: 10.1016/j.radonc.2020.06.029. Epub 2020 Jun 25. PMID: 32593644.
10. Gomez-Iturriaga A, Casquero Ocio F, Ost P, Fernandez I, Rodeño E, Llarena R, Garcia-Olaverri J, Ortiz de Zarate R, Cacicedo J, Ahtamon A, Bilbao P. Outcomes after a first and/or second salvage treatment in patients with oligometastatic prostate cancer recurrence detected by (18-F) choline PET-CT. Eur J Cancer Care (Engl). 2019 Sep;28(5):e13093. doi: 10.1111/ecc.13093. Epub 2019 May 21. PMID: 31115124.
11. Kalinauskaite G, Senger C, Kluge A, Furth C, Kufeld M, Tinhofer I, Budach V, Beck M, Hochreiter A, Grün A, Stromberger C. 68Ga-PSMA-PET/CT-based radiosurgery and stereotactic body radiotherapy for oligometastatic prostate cancer. PLoS One. 2020 Oct 21;15(10):e0240892. doi: 10.1371/journal.pone.0240892. PMID: 33085712; PMCID: PMC7577453.
12. Kroeze SGC, Henkenberens C, Schmidt-Hegemann NS, Vogel MME, Kirste S, Becker J, Burger IA, Derlin T, Bartenstein P, Eiber M, Mix M, la Fougère C, Christiansen H, Belka C, Combs SE, Grosu AL, Müller AC, Guckenberger M. Prostate-specific Membrane Antigen Positron Emission Tomography-detected Oligorecurrent Prostate Cancer Treated with Metastases-directed Radiotherapy: Role of Addition and Duration of Androgen Deprivation. Eur Urol Focus. 2021 Mar;7(2):309-316. doi: 10.1016/j.euf.2019.08.012. Epub 2019 Sep 5. PMID: 31495759.
13. Ost P, Reynders D, Decaestecker K, Fonteyne V, Lumen N, De Bruycker A, Lambert B, Delrue L, Bultijnck R, Claeys T, Goetghebeur E, Villeirs G, De Man K, Ameye F, Billiet I, Joniau S, Vanhaverbeke F, De Meerleer G. Surveillance or Metastasis-Directed Therapy for Oligometastatic Prostate Cancer Recurrence: A Prospective, Randomized, Multicenter Phase II Trial. J Clin Oncol. 2018 Feb 10;36(5):446-453. doi: 10.1200/JCO.2017.75.4853. Epub 2017 Dec 14. PMID: 29240541.
14. Steuber T, Jilg C, Tennstedt P, De Bruycker A, Tilki D, Decaestecker K, Zilli T, Jereczek-Fossa BA, Wetterauer U, Grosu AL, Schultze-Seemann W, Heinzer H, Graefen M, Morlacco A, Karnes RJ, Ost P. Standard of Care Versus Metastases-directed Therapy for PET-detected Nodal Oligorecurrent Prostate Cancer Following Multimodality Treatment: A Multi-institutional Case-control Study. Eur Urol Focus. 2019 Nov;5(6):1007-1013. doi: 10.1016/j.euf.2018.02.015. Epub 2018 Mar 10. PMID: 29530632.
15. Triggiani L, Alongi F, Buglione M, Detti B, Santoni R, Bruni A, Maranzano E, Lohr F, D'Angelillo R, Magli A, Bonetta A, Mazzola R, Pasinetti N, Francolini G, Ingrosso G, Trippa F, Fersino S, Borghetti P, Ghirardelli P, Magrini SM. Efficacy of stereotactic body radiotherapy in oligorecurrent and in oligoprogressive prostate cancer: new evidence from a multicentric study. Br J Cancer. 2017 Jun 6;116(12):1520-1525. doi: 10.1038/bjc.2017.103. Epub 2017 Apr 27.
16. Schick U, Jorcano S, Nouet P, Rouzaud M, Vees H, Zilli T, Ratib O, Weber DC, Miralbell R. Androgen deprivation and high-dose radiotherapy for oligometastatic prostate cancer patients with less than five regional and/or distant metastases. Acta Oncol. 2013 Nov;52(8):1622-8. doi: 10.3109/0284186X.2013.764010. Epub 2013 Apr 2. PMID: 23544357.
